# Characterization of Der f 22 - a paralogue of the major allergen Der f 2

**DOI:** 10.1038/s41598-018-30224-z

**Published:** 2018-08-06

**Authors:** Kavita Reginald, Chye Ling Tan, Simin Chen, Liling Yuen, Sock Yong Goh, Fook Tim Chew

**Affiliations:** 1 Centre for Virus and Vaccine Research, Sunway University, Bandar Sunway, 47500 Selangor Malaysia; 2 Department of Biological Sciences, Sunway University, Bandar Sunway, 47500 Selangor Malaysia; 3 Cell Cycle Control in Skin Epidermis, Institute of Medical Biology, A*STAR, 138648 Singapore, Singapore; 4 Allergy and Molecular Immunology Laboratory, Department of Biological Science, National University of Singapore, 117543 Singapore, Singapore

## Abstract

We previously identified an expressed sequence tag clone, Der f 22, showing 41% amino acid identity to published Der f 2, and show that both genes are possible paralogues. The objective of this study was to characterize the genomic, proteomic and immunological functions Der f 22 and Der f 2. The full-length sequence of Der f 2 and Der f 22 coded for mature proteins of 129 and 135 amino acids respectively, both containing 6 cysteine residues. Phylogenetic analysis of known group 2 allergens and their homologues from our expressed sequence tag library showed that Der f 22 is a paralogue of Der f 2. Both Der f 2 and Der f 22 were single gene products with one intron. Both allergens showed specific IgE-binding to over 40% of the atopic patients, with limited of cross-reactivity. Both allergens were detected at the gut region of *D*. *farinae* by immunostaining. Der f 22 is an important allergen with significant IgE reactivity among the atopic population, and should be considered in the diagnostic panel and evaluated as future hypoallergen vaccine therapeutic target.

## Introduction

*Dermatophagoides farinae* and *D*. *pteronyssinus* are the most important allergy-causing mites worldwide^1^. More than thirty groups of dust-mite allergens have been identified, with the group 1 and 2 being major allergens, causing IgE-responses in about 80% of dust-mite sensitized individuals^2–4^. The identification of new allergens, especially those that show limited cross-reactivity to group 1 and 2 allergens will probably be useful in the diagnosis of allergy in this group of individuals.

We have previously generated an expressed sequence tag (EST) library of *D*. *farinae* to identify the common transcripts in an unbiased fashion^5^. Here, we report the characterization of an IgE-binding protein that showed low homology to previously identified allergens. This allergen was named Der f 22, which was accepted by the World Health Organization (WHO)/International Union of Immunological Societies (IUIS) Sub-committee of Allergen Nomenclature. While Der f 22 showed low linear amino acid sequence identity to the published Der f 2 sequence of 41% (GenBank ID: BAA01241), it shared other characteristics with Der f 2 such as the presence of the lipid-binding ML domain and three-dimensional structural homology as observed by homology modeling, leading us to hypothesize that Der f 22 could be a paralogue of Der f 2.

In this report, we characterize Der f 22 in terms of its gene organization, IgE-binding properties and potential biochemical function, in comparison to Der f 2.

## Results

### Identification and characterization of Der f 22

**Figure 1:**
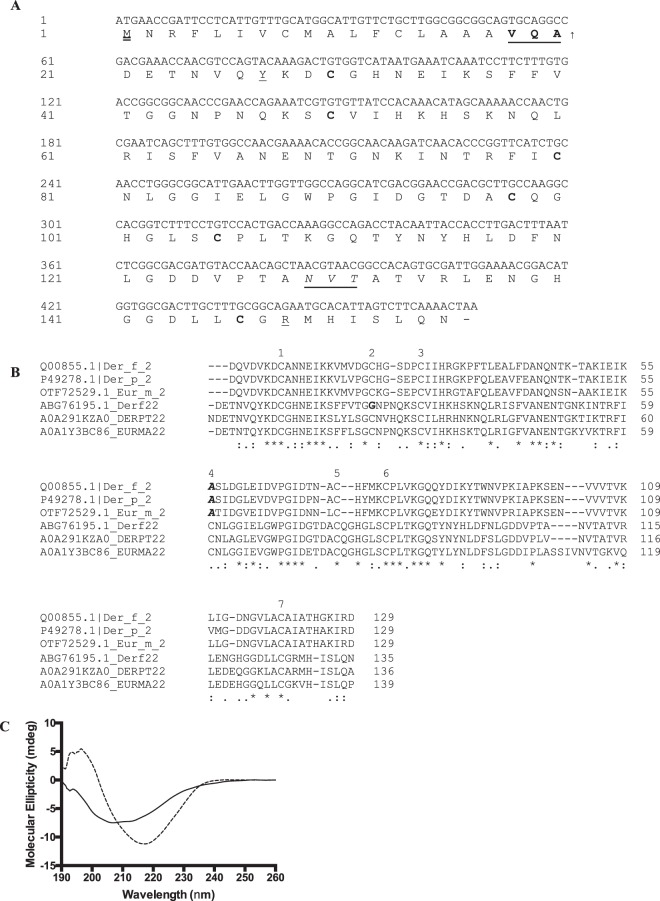
(**A**) Nucleotide and translated amino acid sequence of Der f 22 cDNA. The predicted initiation Met start codon is in double underlined. Underlined and bold-faced amino acids show the predicted signal peptide cleavage site and mature protein starting point. The upward arrow defines the predicted signal peptidase cleavage site. The predicted N-glycosylated site (N-V-T, aa 129-131) is italicized and underlined. Cystein residues along the predicted mature protein sequence are bold faced. The stop codon (TAA) is represented by a dash. The ML domain spans from Y21 to R148 (underlined) of the full length sequence. (**B**) Multiple alignment of mature proteins performed using Clustal O (v1.2.4) using sequences of group 2 allergens (Der f 2, Der p 2 and Eur m 2) and group 22 allergens (Der f 22, Der f 22 homologue protein from *D*. *pteronyssinus*; accession number A0A291KZA0, and Der f 22 homologue protein from *Euroglyphus maynei*; accession number A0A1Y3BC86). Group 22 allergen homologues from *D*. *pterynossinus* and *E*. *maynei* were named as DERPT22 and EURMA22 for simplicity. An asterisk (*) indicated the positions with fully conserved residues, a colon (:) indicated conservation between groups of strongly similar properties, and a period (.) indicated conservation between groups of weakly similar properties. Numbers above the sequences indicate the presence of a cysteine residue in any of the proteins. The glycine (**G**) residue of mature Der f 22 at position 2 is in bold, whereas the alanine (**A**) residues in group 2 allergens at position 4 are bold and italicized. (**C**) CD spectra of Der f 22 (solid line) and Der f 2 (dotted line). Twenty micromolar of each protein, in 50 mM sodium acetate pH 4.6 were used to obtain the circular dichroism spectra. Spectra shown are averages of 10 scans. Der f 2 shows a spectrum of a typical β-sheeted protein with minima at 218 nm, whereas Der f 22 does not.

We identified an EST clone from our *D*. *farinae* library^5^ with a low (41%) but significant homology to Der f 2 as observed by its three-dimensional structural homology and Pfam domain classification. This antigen was a potential allergen as preliminary screens showed that it could bind to IgE present in sera of atopic individuals. This clone was named Der f 22, in accordance to the allergen nomenclature guidelines (WHO/IUIS, 1994), and its sequence deposited in Genbank (DQ643992.1) in June 2007. The complete clone of Der f 22 had 468 nucleotides, encoding for 155 amino acids (Fig. 1A). Der f 22 has a predicted signal peptide with a cleavage site between residues 20 and 21, VQA-DE, resulting in a 135 amino acid mature protein. The mature Der f 22 protein was 6 amino acids longer than Der f 2, and the pro-peptide sequence of Der f 22 (20 amino acids) was longer than Der f 2 by three residues. Der f 22 had one predicted N-glycosylation site at residues 129–131 (N-V-T), predicted by the NetNGlyc algorithm^6^, which was absent in Der f 2 or other group 2 allergens.

Database search using Der f 22 as the query sequence revealed the presence of two other Der f 22-like sequences from *D*. *pteronyssinus*^7^ (78% amino acid sequence identity) and *Euroglyphus maynei* (79% identity). The Der f 22 homologue protein from *E*. *maynei* had a signal peptide of 21 amino acids, while that from *D*. *pteronysinnus* was 28 amino acids long. Based on the multiple sequence alignment, all group 2 allergens (Der f 2, Der p 2 and Eur m 2) has six cysteine residues (Fig. 1B), which are known to form three disulfide bonds based on structural studies^8,9^ and appear to be critical in maintaining the three dimensional (3D) structure of the protein^10^. However, the arrangement of cysteine residues differed in Der f 22 and its homologous proteins when compared to group 2 allergens. While Der f 22 also had six cysteine residues, their location differed from Der f 2 in two positions. First, at position 2, Der f 22 has a glycine instead of cysteine residue and at position 4, Der f 22 has a cysteine residue while Der f 2 has an alanine at the same position (Fig. 1B). It is worthy to note the cysteine residue at position 4 was conserved in Der f 22 and its homologues while the group 2 allergens maintained an alanine residue.

Circular dichroism spectra of Der f 22 was shifted from that of a typical β-sheeted structure of Der f 2 (Fig. 1C), which could reflect some differences in the 3D structure of both proteins. Curiously, Der f 22-homologues from *D*. *pteronyssinus* and *E*. *maynei* have seven cysteine residues. As the cysteine residues in Der f 22 are positioned differently compared to that of Der f 2, there is a possibility that some cysteines may not be involved in disulfide bond formations due to the distances between the cysteine pairs. Similarly, in Der f 22-homologues, the presence of seven cysteine residues would result in at least one free cysteine residue. The presence of molecules with free cysteine residues could lead to the formation of homo- or heterodimers by disulfide bond linkages, which could eventually alter the allergenicity of the molecule.

Analysis using the Pfam software^11^ revealed that Der f 22 contained the Der-p2_like domain, which is a member of the ML (MD-2-related Lipid-recognition) domain family. This was a common feature of other group 2 allergens, and has been identified in several other proteins, including the Niemann-Pick type C2 (NPC2) protein, which is a structural homologue of Der f 2. The ML domain is predicted to be involved in lipid binding, and its structure is characterized by two anti-parallel β-pleated sheets and an accessible central hydrophobic cavity.

### Genomic organization of Der f 22 and Der f 2

Both Der f 22 and Der f 2 had one intron, with varied positions. The intron of Der f 2 was located at the 5′ end of the gene^12^, with a 90-base pair type 1 intron interrupting the codon at amino acid residue 9 of the mature protein (Supplementary Fig. S1A). In contrast, the intron of Der f 22 was positioned at the 3′ end and consists of a type 0 intron of 78 base pairs, located between codons 127 and 128 of the mature protein (Supplementary Fig. S1B). For both genes, the intron-exon splice junction sequence followed the GT-AG rule^13^. Substitutions at twelve nucleotides were observed when comparing cDNA and gDNA sequences of Der f 2, which are likely due to polymorphisms, a common phenomenon observed in group 2 allergens^14–16^.

### Southern blot analysis

Southern blot was performed using genomic DNA of *D*. *farinae* digested with four restriction enzymes (RE), EcoR I, Msc I, Hind III and BamH I. None of the restriction enzyme sites were present in the complete sequence of Der f 2, whereas for Der f 22, Msc 1 restriction site was present 116 bp upstream of the hybridization probe. A single labeled band was observed in each restriction enzyme reaction for both Der f 22 and Der f 2 (Supplementary Fig. S2), showing that both genes were present as a single gene copy. Each of these genes were located at different loci on the mite genome, as the banding pattern of the RE-digested genomic DNA differed.

### IgE-binding capacities of Der f 22 and Der f 2

**Figure 2:**
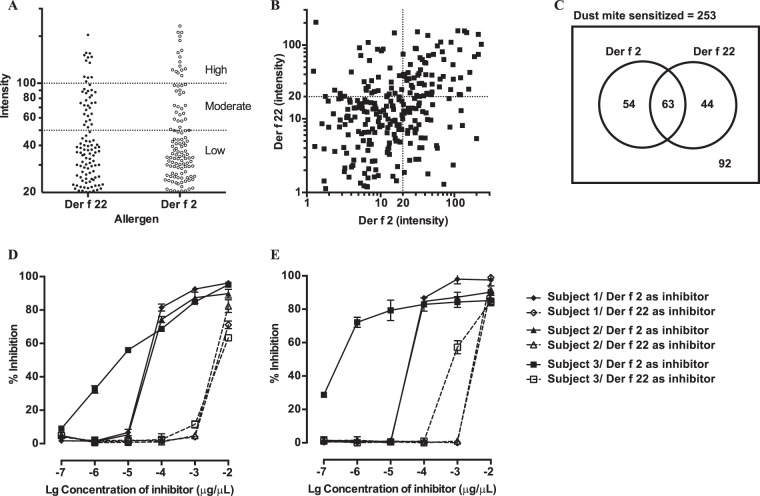
IgE-binding capacity of Der f 22 and Der f 2. (**A**) Scatter plot of IgE-binding of atopic individuals. The amount of IgE-binding was measured as optical density. (**B**) Dot-plot of IgE-binding of atopic patients’ sera to Der f 22 and Der f 2 shows that the IgE-binding intensities between both allergen are correlated (r^2^ = 0.2337, p < 0.0001). (**C**) Venn diagram depicting the number of individuals with IgE-reactions to recombinant Der f 2 and Der f 22 as measured using immuno-dot blots. (**D**,**E**) Competitive IgE ELISA assay. Three individual sera (♦ subject 1, ▲ subject 2, ■ subject 3) were pre-absorbed with various concentrations of inhibitors before incubation with (**C**) Der f 2 or (**D**) Der f 22 in coated plates. Filled symbols and open symbols indicate Der f 2 and Der f 22 respectively, were used as the inhibitor.

Sera specific IgE responses of 253 dust-mite sensitized individuals to Der f 22 and Der f 2 was measured using immuno-dot blot assay (Fig. 2A). Both allergens showed IgE-binding to approximately the same proportion of the population with about 46% displaying IgE reactions to Der f 2 and 42% to Der f 22. The number of individuals with high IgE-binding to Der f 2 (intensity >100, equivalent to Class 3 specific IgE levels) was only slightly higher than Der f 22 (16 versus 12 individuals). Sixty-three individuals had serum IgE-binding to both allergens, of which 10 showed high IgE-binding. There was no significant difference in the mean serum IgE binding intensities to both allergens (p = 0.2738). IgE binding levels between Der f 2 and Der f 22 were correlated (r^2^ = 0.2337, p < 0.0001; Fig. 2B). This could indicate that patients with IgE-reactions to both Der f 2 and Der f 22 could be due to either co-sensitization or cross-reactivity. The patient cohort tested shows that some of them are mono-sensitised to either allergen, react to both allergens (n = 63), or do not react to either allergen (Fig. 2C).

We next assayed the IgE cross-reactivity between Der f 2 and Der f 22 by inhibition ELISA, using sera from three individuals with high IgE-binding to both allergens, and from whom sufficient sera was available. Both allergens displayed limited cross-reactivity (Fig. 2D,E). For all sera tested, a concentration of 0.05 mg/mL of allergen was sufficient for self-inhibition (>90%). Control experiments using BSA as a non-specific inhibitor showed no inhibitory effect (data not shown).

### Immunostaining of Der f 22 and Der f 2 on sectioned D. farinae

**Figure 3:**
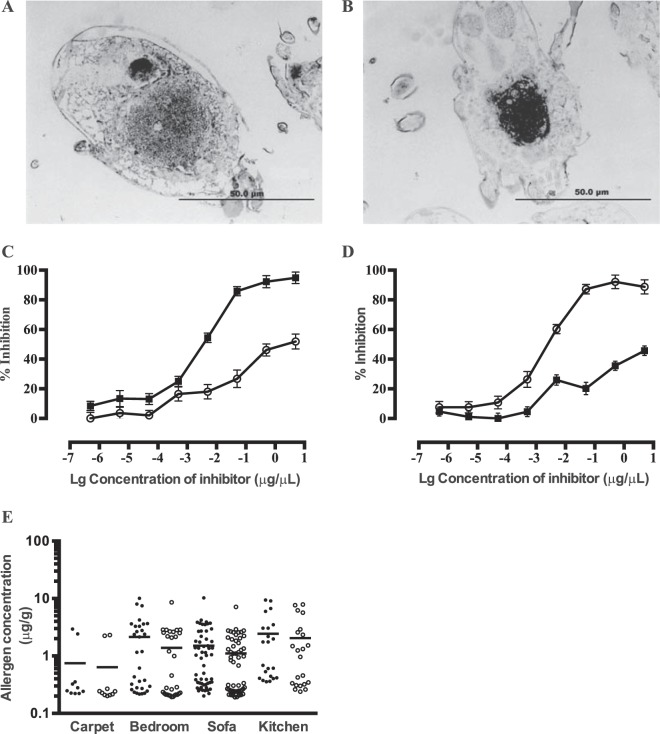
Microtome-cut *D*. *farinae* sections probed with rabbit polyclonal against Der f 2 (**A**) and Der f 22 (**B**). Sections were viewed using light microscope with 200X magnification. Areas of positive staining (arrows) of anterior midgut (Amg) and hindgut (Hg) are indicated. (**C**,**D**) Competitive IgG ELISA assay with polyconal antibodies generated from rabbits. Anti -Der f 22 IgG (○) and anti-Der f 22 IgG (■) were inhibited with various concentrations of inhibitors before incubation with (**C**) Der f 2 or (**D**) Der f 22 in coated plates. Filled symbols and open symbols indicate Der f 2 and Der f 22 respectively, were used as the inhibitor. (**E**) Concentration of Der f 22 (●) and Der f 2 (○) in dust samples were assayed using ELISA. Dust samples were collected by vacuuming four areas within a home, carpet (n = 10), bedroom (n = 31), sofa (n = 56) and kitchen (n = 22). Horizontal bars indicate the mean concentration of allergen in each sample.

Immunostaining of *D*. *farinae* sections showed that Der f 2 stained mainly the hindgut and to a lesser extent the anterior mid gut (Fig. 3A). In contrast, Der f 22 was strongly localized at the anterior mid gut region (Fig. 3B). At the concentrations used for immunostaining, both pAbs displayed low cross-reactivity, ensuring specific stainings (Fig. 3C,D).

### Presence of Der f 22 and Der f 2 in the indoor environment

The dust samples from four niches (carpet, bedroom, sofa and kitchen) of 56 homes of individuals with house dust-mite allergy were assayed for the levels of Der f 2 and Der f 22 (Fig. 3E). The concentration of Der f 22 was highest in the kitchen and lowest in the carpet with an average of 2.44 μg/g and 0.75 μg/g respectively. Similarly, the highest levels of Der f 2 was detected in the kitchen, averaging 2.04 μg/g and lowest in the carpet with an average of 0.64 μg/g. On the whole, the levels of Der f 22 was about 1.4-folds higher compared to Der f 2 in the indoor environment.

### Presence of paralogues in other dust-mite group 2 allergens

**Figure 4:**
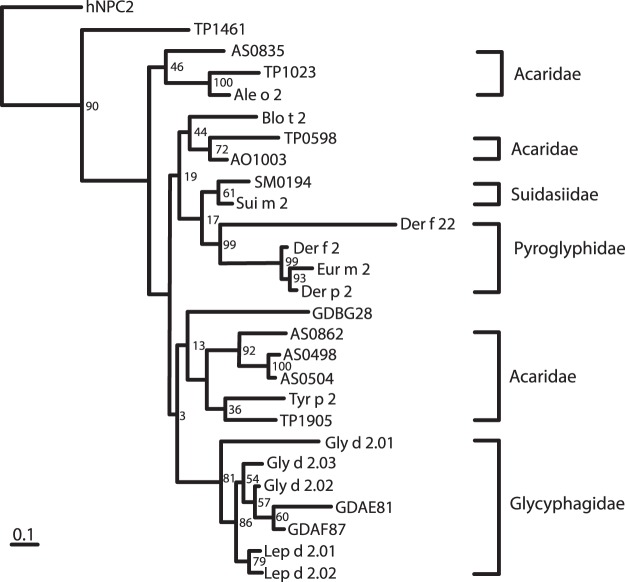
Phylogenetic relationships of known and putative group 2 allergens. All sequences showed homology to group 2 allergens (E-value < 0.001) when searched using BLAST-X algorithm. The phylogenetic tree was generated with PHYML using the maximum-likelihood method. Numbers on branches indicate bootstrap values (100 simulations). Tree was rooted with hNPC2 sequence, which was used as the outgroup. Some sequence names in the tree were shortened: Gly d 201 (Gly d 2.01), Gly d 202 (Gly d 2.02), Gly d 203 (Gly d 2.03), Lep d 201 (Lep d 2.01) and Lep d 202 (Lep d 2.02).

Based on the phylogenetic tree, Der f 22 clustered in the same clade as Der f 2, and other group 2 allergens from the Pyroglyphidae family (Fig. 4). The longer branch length of Der f 22 indicates that it has more nucleotide substitutions compared to other members of that clade. The event of paralogy most likely took place after speciation, as Der f 22 and Der f 2 were clustered in the same clade.

Similar to the Pyroglyphidae family, the gene duplication event for group 2 allergen from the Suidasiidae and Glycyphagidae families also likely occurred after speciation. For other group 2 allergens, gene duplication events could have occurred before speciation. Group 2 allergen homologues from the Acaridae family *(Tyrophagus putrescentiae*, *Acarus siro* and *Aleuroglyphus ovatus*) clustered in three separate clades, suggesting that two gene duplication events had occurred prior to speciation. In the first clade of Acaridae family, sequences from *A*. *siro*, *T*. *putrescentiae* and *A*. *ovatus* were clustered together. However, in the second and third Acaridae clusters, only two of the three putative allergen sequences were present, possibly due to incomplete data, or loss in evolution.

Two sequences, GDBG28 (from *G*. *domesticus*) and TP1461 (from *T*. *putrescentiae*) were very divergent from other sequences originating from the same species, and could not be accurately placed in the right clade (Fig. 4). The phylogenic tree suggests that the event of paralogy was common for this group of proteins (Fig. 4).

## Discussion

We report the genomic, proteomic and functional characterization of Der f 22 identified from *D*. *farinae*^5^. The mature Der f 22 protein had 135 amino acids with six cysteine residues and a signal peptide, a feature that is shared by all group 2 mite allergens. However, the positions of the cysteine residues of Der f 22 were different from that of Der f 2 and other group 2 allergens. Even when compared to other Der f 22-homologue proteins, there were differences, mainly in the number of cysteine residues, as the homologues had seven cysteine residues. Additional investigations of the molecular size of Der f 22 under non-reducing and non-denaturing conditions, three-dimensional structure solution or cysteine mutation studies would be very valuable to clearly define the disulfide bonding pattern, and to evaluate if this protein is able to form homo- or hetero- dimers in the event that unpaired cysteine residues are present. The formation of dimers could alter the allergenicity of the molecule in terms of accessibility of IgE to its epitopes and IgE cross-linking, as observed in previous studies involving trimers of the major birch pollen allergen, Bet v 1^17^.

Recently, another group reported the isolation of Der f 22 from mite RNA using primer sequences designed based on our initial GenBank submission^18^. While Der f 22 and Der f 2 share many similar characteristics, some key differences were also present. The signal peptide in Der f 22 (20 amino acids) is 3 amino acids longer than that of Der f 2 and other group 2 dust-mite allergens (range: 15–17 amino acids). The genome organization of Der f 22 was similar to that of Der f 2, in terms of the number of introns and exons. Curiously, the intron size of Der f 22 (78 bp) showed more similarity to that of Lep d 2.01 (76 bp) and Lep d 2.02 (75 bp)^19^ as compared to Der f 2 (90 bp)^12^. However, the intron of the Der f 22 gene is located further downstream compared to Der f 2, Lep d 2.01 and Lep d 2.02. Southern blot hybridization showed that both genes encoding for Der f 22 and Der f 2 were from a single gene family and located at different loci on the mite genome. The Der f 22 allergen could be detected in the indoor environment, with average concentrations at least 1.4-fold higher than Der f 2 in all niches assessed in this study.

Der f 22 is an important allergen as it was able to bind sera IgE in about 42% of allergic individuals with limited cross-reactivity to Der f 2, indicating that Der f 22 contained unique IgE epitopes. It has been demonstrated that the 3D structure of group 2 allergens, maintained primarily by their disulfide bonds, are important in maintaining the IgE epitopes. Mutations to cysteine residues resulted in protein structural changes and caused the IgE-binding capacity to reduce^20,21^. Studies on the structural solution together with IgE-epitope mapping of Der f 22 would provide more insights on the links between the influence of protein structure on the IgE binding capacity of Der f 22.

Both Der f 22 and Der f 2 were found to be localized at the gut, although the specific region varied. Der f 22 concentrated exclusively at the anterior midgut region, while Der f 2 was found at higher concentrations at the hindgut, while also present at lower concentrations in other organs. While it is not clear why both proteins are localized at the gut region, it is tempting to speculate that this could be related to the lipid binding property of the proteins which is a feature of the ML domain family.

Paralogue gene families are results of gene duplication, and are thought to be retained in an organism because of selective benefits. Based on several observations, Der f 22 is likely to be a putative paralogue of Der f 2. First, both proteins belong to the same ML domain family. Second, both proteins share high structural homology albeit low linear sequence identity. Third, Der f 22 and Der f 2 showed similar IgE-binding capacities with limited cross-reactivity between them.

Due to sequence divergence between paralogous genes, their discovery using standard molecular techniques can be difficult and time consuming. Using the expressed sequence tag (EST) approach^5^, we identified several putative paralogues in different dust-mite allergen groups, such as Blo t 21 as a paralogue of Blo t 5^22^, and several families of group 7 and 13 paralogues allergens^5^, indicating the frequent occurrence of paralogous sequences in the dust-mite genome. Recently, paralogues of a major strawberry allergen, Fra a 1 has also been identified based on genomic databases^23^, expanding the identification of paralogues beyond dust mite allergens. The identification and understanding of paralogue allergens are definitely important for designing good diagnostics and therapeutics for allergies.

Der f 22 is an important dust-mite allergen as it has significant, but incomplete cross-reactivity to its paralogue Der f 2. In view of improving diagnosis of allergies, the addition of Der f 22 to the allergen test panel is recommended. Three-dimensional structural characterization and IgE-epitope mapping of Der f 22 would be critical for downstream applications such as the generation of hypoallergen vaccine candidates for immunotherapy.

## Materials and Methods

### Genomic DNA extraction and Southern Blot analysis

Fifty milligrams of cultured *D*. *farinae* (Central Science Laboratories, UK) were homogenized with liquid nitrogen and genomic DNA (gDNA) extracted using the DNeasy® kit (Qiagen). A DNA segment of Der f 2 (217 bp) and Der f 22 (204 bp) was PCR-amplified and labeled with digoxigenin (DIG) using PCR DIG Probe Synthesis Kit (Roche Diagnostics) as the hybridization probe. Six micrograms of *D*. *farinae* gDNA was digested to completion using *Eco*R I, *Hin*d III and *Bam*H I and *Msc* I restriction enzymes and separated by electrophoresis. The DNA was denatured, transferred to a nylon membrane (Hybond-N + , Amersham International), and hybridized with specific DIG labeled probes. DIG luminescent detection kit (Roche Diagnostics) was used as the detection system.

### Isolation of Genomic Der f 2 and Der f 22 Genes

Genomic clones of Der f 2 and Der f 22 were isolated by PCR with 1 μg of *D*. *farinae* gDNA using primer pairs detailed in supplementary data. The amplified products were ligated into pGEM-T Easy vector (Promega) and transformed into *E*. *coli* DH5-α competent cells. Plasmids were purified using QIAprep® Spin Miniprep Kit (Qiagen), and sequenced.

### Expression of recombinant proteins, CD spectra, and antibody production

DNA encoding the mature transcript of Der f 2 (DQ643992) and Der f 22 (Q00855) were amplified by PCR, ligated into a modified pET-32a (Novagen) and transformed into *Eschericia coli* strain BL21(DE3). Recombinant proteins were expressed as a His-tagged soluble protein with 1 mM IPTG induction, purified using Ni-NTA resin (Novagen) and refolded by rapid dilution in 50 mM sodium acetate, pH 4.6. Circular dichroism (CD) spectra was performed as previously described^24^ using 10 μM of protein. Polyclonal antibodies (pAb) against Der f 2 and Der f 22 were raised in New Zealand White rabbits by subcutaneous injection with 300 μg of recombinant protein with Freund’s adjuvant. Monoclonal antibodies (mAb) against Der f 2 and Der f 22 were prepared from Balb/c immunized mice using the myeloma fusion method^25^.

### Ethics approval for serum samples and animal immunizations

Consecutive serum samples from patients from Singapore with clinical symptoms of allergies were used. Written informed consents were obtained from all participants (n = 253, age range 13–55 years old, mean age = 24 years old) to obtain blood samples. For participants under the age of 18 years, informed consent were obtained from a parent and/or legal guardian. Inclusion and exclusion criteria for selecting patients for this study are detailed in Supplementary Table S1. The human and animal studies were reviewed and approved by the Institutional Review Board of the National Healthcare Group Domain Specific Review Board - B/04/055, National University of Singapore (NUS) Institutional Review Board: 07–023, 09–256, 10–445, 13–075 and B14–150 and the Animal Research Ethics Committee of NUS, and are in compliance with the Helsinki declaration.

### Immuno-dot blot

Immuno-dot blot assays were performed as previously described^24,26–29^. Briefly, 1 μg of recombinant protein was dotted on nitrocellulose membranes. Series-diluted IgE standards (National Institute for Biological Standards, UK) was used as positive control, and bovine serum albumin (BSA) as negative. Spot intensities were measured using an imaging software (Microimage v.3.01) and were recorded after subtrating the local background. Intensities >20 (equivalent to 2 SDs above the mean negative sera responses) were considered positive. Inter- and intra-assay concordance exceeded 90% and 95%, respectively, demonstrating strong assay reproducibility. Multiple dilution experiments were performed to demonstrate linear parallelism between the specific IgE and total IgE standard curves over the linear range of the specific IgE dilutions.

### Inhibition ELISA

ELISA plates (NUNC) were coated overnight with 250 ng protein. The plates were blocked with PBS-T (0.1%) and incubated with sera pre-absorbed with varied amounts of recombinant Der f 2 or Der f 22 overnight. Biotin conjugated anti-human IgE mAb (BD-Pharmingen) was added for 2 hours, followed by avidin-HRP (BD-Pharmingen) for 30 mins. After washing, 100 μL of 3,3′,5,5′-Tetramethylbenzidine (TMB; Sigma) was added per well. The reaction was stopped with 20 μL 1 M HCl, and absorbance measured at 450 nm using a micro-plate reader.

### Immunostaining

Live *D*. *farinae* mites were prepared as previously described^28^ except that 10 μm microtome sections were used and immunostaining was performed using pAb against Der f 2 or Der f 22. Organ identification and terminologies used were as previously reported^30^.

### Dust sample collection and processing

Dust samples were collected from different niches of 56 homes in Singapore using a modified Kirby Classic III vacuum cleaner (Kirby) adapted with a chamber that collects dust onto filter paper. Sample were obtained by vacuuming an area of 1 m^2^ for 2 mins, sieved (500 μm pore size), mixed with PBS (1 mL PBS for 50 mg dust sample), and agitated overnight at 4 °C. The samples were centrifuged at 2500 rpm for 20 mins at 4 °C, and the supernatants were stored at −20 °C.

### Measurement of allergen amounts in dust samples

One hundred microliters of individual dust sample were coated overnight onto wells pre-coated with the Der f 2 or Der f 22 specific capture mAb. The wells were washed with PBS-T (0.05%) between each step. Wells were blocked with PBS-BSA (1%) for 30 mins followed by overnight incubation with 100 μL rabbit anti-Der f 2 or anti-Der f 22 IgG pAb at 1:5000 dilutions in PBS. Wells were then incubated with anti-rabbit IgG-conjugated horseradish peroxidase (BD Pharmingen) in PBS for 3 hours. Finally, TMB was added for 30 min, the reaction stopped using 1 M HCl (20 μL) and plates were read at 450 nm.

### Phylogenetic analysis

Nucleotide sequences with homology to group 2 allergens (from nine dust-mite species) were obtained via BLAST-X search (E-value < 0.001) from both public and our EST sequences. Nucleotide sequences were aligned using SequenceHelper, with manual editing to optimize alignments. The phylogenetic tree was generated with PHYML using the maximum-likelihood method (bootstrap = 100). The tree was rooted with hNPC2 sequence, which was used as the outgroup. The GenBank ID numbers for the sequences used are detailed in supplementary data.

### Statistical analysis

Statistical analysis was performed using GraphPad Prism version 6.0. Differences in serum IgE binding and allergen levels in the dust samples were performed using unpaired t-test (p < 0.05, two tailed). Correlations were calculated with Pearson’s correlation test at 95% confidence interval.

## Electronic supplementary material

Supplementary Information

## References

[1] Thomas WR, Smith WA, Hales BJ (2004). The allergenic specificities of the house dust mite. Chang Gung Med J.

[2] Thomas WR (2015). Hierarchy and molecular properties of house dust mite allergens. Allergol Int.

[3] Platts-Mills TA, Vervloet D, Thomas WR, Aalberse RC, Chapman MD (1997). Indoor allergens and asthma: report of the Third International Workshop. J Allergy Clin Immunol.

[4] Thomas WR, Smith W (1998). House-dust-mite allergens. Allergy.

[5] Angus AC, Ong ST, Chew FT (2004). Sequence tag catalogs of dust mite-expressed genomes: utility in allergen and acarologic studies. Am J Pharmacogenomics.

[6] Gupta, R. J., E.; Brunak, S. Prediction of N-glycosylation sites in human proteins. *In Preparation* (2004).

[7] Bordas-Le Floch V (2017). A combined transcriptome and proteome analysis extends the allergome of house dust mite Dermatophagoides species. PLoS One.

[8] Ichikawa S (2005). NMR study on the major mite allergen Der f 2: its refined tertiary structure, epitopes for monoclonal antibodies and characteristics shared by ML protein group members. J Biochem.

[9] Derewenda U (2002). The crystal structure of a major dust mite allergen Der p 2, and its biological implications. J Mol Biol.

[10] Mueller GA, Smith AM, Chapman MD, Rule GS, Benjamin DC (2001). Hydrogen exchange nuclear magnetic resonance spectroscopy mapping of antibody epitopes on the house dust mite allergen Der p 2. J Biol Chem.

[11] Bateman A (2004). The Pfam protein families database. Nucleic Acids Res.

[12] Yuuki T, Okumura Y, Okudaira H (1997). Genomic organization and polymorphisms of the major house dust mite allergen Der f2. Int Arch Allergy Immunol.

[13] Mount SM (1982). A catalogue of splice junction sequences. Nucleic Acids Res.

[14] Smith AM (2001). Themolecular basis of antigenic cross-reactivity between the group 2 mite allergens. J Allergy Clin Immunol.

[15] Piboonpocanun S, Malainual N, Jirapongsananuruk O, Vichyanond P, Thomas WR (2006). Genetic polymorphisms of major house dust mite allergens. Clin Exp Allergy.

[16] Jeong KY (2012). Sequence polymorphisms of Der f 1, Der p 1, Der f 2 and Der p 2 from Korean house dust mite isolates. Exp Appl Acarol.

[17] Campana R (2011). Altered IgE epitope presentation: A model for hypoallergenic activity revealed for Bet v 1 trimer. Mol Immunol.

[18] Cui YB (2015). TheDermatophagoides farinae group 22 allergen: cloning and expression in Escherichia coli. Int Forum Allergy Rhinol.

[19] Kaiser L, Gafvelin G, Johansson E, van Hage-Hamsten M, Rasool O (2003). Lep d 2 polymorphisms in wild and cultured Lepidoglyphus destructor mites. Eur J Biochem.

[20] Takai T (2000). Unlocking the allergenic structure of the major house dust mite allergen der f 2 by elimination of key intramolecular interactions. FEBS Lett.

[21] Nishiyama C (1995). Analysis of the IgE-epitope of Der f 2, a major mite allergen, by *in vitro* mutagenesis. Mol Immunol.

[22] Tan KW (2012). NMR structure and IgE epitopes of Blo t 21, a major dust mite allergen from Blomia tropicalis. J Biol Chem.

[23] Ishibashi M (2018). Analysis of major paralogs encoding the Fra a 1 allergen based on their organ-specificity in Fragaria x ananassa. Plant Cell Rep.

[24] Chan SL, Ong ST, Ong SY, Chew FT, Mok YK (2006). Nuclear magnetic resonance structure-based epitope mapping and modulation of dust mite group 13 allergen as a hypoallergen. J Immunol.

[25] Shen HD (1996). IgE and monoclonal antibody binding by the mite allergen Der p 7. Clin Exp Allergy.

[26] Kidon MI (2011). Mite component-specific IgE repertoire and phenotypes of allergic disease in childhood: the tropical perspective. Pediatr Allergy Immunol.

[27] Chan SL (2008). Nuclear magnetic resonance structure and IgE epitopes of Blo t 5, a major dust mite allergen. J Immunol.

[28] Gao YF (2007). Identification and characterization of a novel allergen from Blomia tropicalis: Blo t 21. J Allergy Clin Immunol.

[29] Batard T (2006). Production and proteomic characterization of pharmaceutical-grade Dermatophagoides pteronyssinus and Dermatophagoides farinae extracts for allergy vaccines. Int Arch Allergy Immunol.

[30] Brody ARMJC, Wharton GW (1972). Dermatophagoides farinae: The Digestive System. J. N. Y. Entomol. Soc..

