# Supplementary material for: Characterization of Der f 22 - a paralogue of the major allergen Der f 2
Source: Sci Rep. 2018 Aug 6;8:11743. doi: 10.1038/s41598-018-30224-z (PMC6079044; doi:10.1038/s41598-018-30224-z)
Supplement: Supplementary file 1 — Supplementary Information [file 41598_2018_30224_MOESM1_ESM.pdf]

Characterization of Der f 22 - a paralogue of the major allergen Der f 2

Short title: Characterization of an allergen, Der f 22

Kavita REGINALD[1,2], Chye Ling TAN[3], Simin CHEN[4], Liling YUEN[4], Sock Yong GOH[4] and Fook Tim CHEW[4*]

[1]Centre for Virus and Vaccine Research, Sunway University, Bandar Sunway 47500, Selangor, Malaysia

[2]Department of Biological Sciences, Sunway University, Bandar Sunway 47500, Selangor, Malaysia

[3]Cell Cycle Control in Skin Epidermis, Institute of Medical Biology, A*STAR, 138648 Singapore

[4]Allergy and Molecular Immunology Laboratory, Department of Biological Science, National University of Singapore, 117543 Singapore

Corresponding author:

Dr. Chew Fook Tim, Allergy and Molecular Immunology Laboratory, Lee Hiok Kwee Functional Genomics Laboratories, Department of Biological Sciences, 14 Science Drive 4, National University of Singapore, 117543 Singapore, Tel. +65-65161685; Fax +65-67792486; E-mail: dbscft@nus.edu.sg

Supplementary Data

Genomic clones of Der f 2 and Der f 22 were isolated by PCR with 1μg of genomic *D. farinae* DNA using the following primer pairs: Der f 2 forward (5'-ATGATTTCCAAAATTTGTGC-3') and reverse (5'-TTAATCACGCATTTTAGCGTG-3') primers, and Der f 22 (5'-ATGAACCGATTCCTCATTGTT-3') and reverse (5'-CCGGAATTCCGGTTAGTTTT GAAGACT-3') primers.

The GenBank accession numbers for the sequences used were: hNPC2 (AAH02532), TP1461 (CN767356), AS0835 (DR075304), TP1023 (CN767227), Ale o 2 (AAS75832), Blo t 2 (DQ677253), TP0598 (CN766836), AO1003 (CN765143), SM0194 (CN737971), Sui m 2 (AAS75831), Der f 22 (DQ643992), Der f 2 (Q00855), Eur m 2 (Q9TZZ2), Der p 2 (DQ677264), GDBG28 (CO437347), AS0498 (DR075197), AS0504 (DR075200), Tyr p 2 (CAA73221), TP1905 (CN767658), Gly d 2.01(Q9U5P7), Gly d 2.03 (AAQ54603), Gly d 2.02 (CAB76459), GDAE81 (CO438261), GDAF87 (CO438634), Lep d 2.01 (P80384), Lep d 2.02 (S66499).

Supplementary Table S1. Inclusion and exclusion criteria for patients used in this study

Inclusion criteria	
	Singaporean citizen
	Doctor diagnosed allergy and/or allergic rhinitis
	Positive skin prick results and/ or had IgE binding to dust-mite crude protein extracts (*Dermatophagoides spp.* and/or *Blomia tropicalis*) as tested using immuno-dot blots
Exclusion criteria	
	Not a Singaporean citizen
	Negative skin prick results and/ or had IgE binding to dust-mite crude protein extracts (*Dermatophagoides spp.* and/or *Blomia tropicalis*) as tested using immuno-dot blots

```
A   Der f 2 cDNA    ATGATTTCCAAAATCTTGTGCCTTTCATTGTTGGTAGCAGCCGTTGTTGCCGATCAAGTC
    Der f 2 gDNA    ATGATTTCCAAAATCTTGTGCCTTTCATTGTTGGTAGCAGCCGTTGTTGCCGATCAAGTC
                    ************************************************************

    Der f 2 cDNA    GATGTTAAAGATTGTG--------------------------------------------
    Der f 2 gDNA    GATGTTAAAGATTGTGGTAAGTTTGTTTGTGTGATACATTTTTTTTTTCTTTTCATTTAT
                    ****************

    Der f 2 cDNA    ----------------------------------------------CCAACAATGAAATC
    Der f 2 gDNA    TCAACACTAAACACAATTTTCTTTTTTATTGTTTATATTCTTATAGCCAACAATGAAATC
                                                                  **************

    Der f 2 cDNA    AAAAAAGTAATGGTCGATGGTTGCCATGGTTCTGATCCATGCATCATCCATCGTGGTAAA
    Der f 2 gDNA    AAAAAAGTAATGGTCGATGGTTGCCATGGTTCTGATCCATGCATCATCCATCGTGGTAAA
                    ************************************************************

    Der f 2 cDNA    CCATTCACTTTGGAAGCCTTATTCGATGCCAACCAAAACACTAAAACCGCTAAAATTGAA
    Der f 2 gDNA    CCATTCACTTTGGAAGCCTTATTCGATGCCAATCAAAACACTAAAACCGCTAAAATTGAA
                    ********************************* **************************

    Der f 2 cDNA    ATCAAAGCCAGCCTCGATGGTCTTGAAATTGATGTTCCCGGTATCGATACCAATGCTTGC
    Der f 2 gDNA    ATCAAAGCTAACATCAATGGTCTTGAAGTTGATGTTCCCGGTATCGATACCAATGCTTGC
                    ******** * * ** *********** ********************************

    Der f 2 cDNA    CATTTTATGAAATGTCCATTGGTTAAAGGTCAACAATATGATATCAAATATACATGGAAT
    Der f 2 gDNA    CATTATATCAAATGTCCATTGGTTAAAGGTCAACAATATGATGCCAAATATACATGGAAT
                    **** *** *********************************  ****************

    Der f 2 cDNA    GTGCCGAAAATTGCACCAAAATCTGAAAACGTTGTCGTTACAGTCAAACTTATCGGTGAT
    Der f 2 gDNA    GTACCGAAAATTGCACCAAAATCTGAAAACGTTGTCGTTACAGTCAAACTTATCGGTGAT
                    ** *********************************************************

    Der f 2 cDNA    AATGGTGTTTTGGCTTGCGCTATTGCTACCCATGGTAAAATCCGTGATTAA
    Der f 2 gDNA    AATGGTGTTTTGGCTTGCGCTATTGCTACCCACGCTAAAATCCGTGATTAA
                    ******************************** * ****************


B   Der f 22 cDNA   ATGAACCGATTCCTCATTGTTTGCATGGCATTGTTCTGCTTGGCGGCGGCAGTGCAGGCC
    Der f 22 gDNA   ATGAACCGATTCCTCATTGTTTGCATGGCATTGTTCTGCTTGGCGGCGGCAGTGCAGGCC
                    ************************************************************

    Der f 22 cDNA   GACGAAACCAACGTCCAGTACAAAGACTGTGGTCATAATGAAATCAAATCCTTCTTTGTG
    Der f 22 gDNA   GACGAAACCAACGTCCAGTACAAAGACTGTGGTCATAATGAAATCAAATCCTTCTTTGTG
                    ************************************************************

    Der f 22 cDNA   ACCGGCGGCAACCCGAACCAGAAATCGTGTGTTATCCACAAACATAGCAAAAACCAACTG
    Der f 22 gDNA   ACCGGCGGCAACCCGAACCAGAAATCGTGTGTTATCCACAAACATAGCAAAAACCAACTG
                    ************************************************************

    Der f 22 cDNA   CGAATCAGCTTTGTGGCCAACGAAAACACCGGCAACAAGATCAACACCCGGTTCATCTGC
    Der f 22 gDNA   CGAATCAGCTTTGTGGCCAACGAAAACACCGGCAACAAGATCAACACCCGGTTCATCTGC
                    ************************************************************

    Der f 22 cDNA   AACCTGGGCGGCATTGAACTTGGTTGGCCAGGCATCGACGGAACCGACGCTTGCCAAGGC
    Der f 22 gDNA   AACCTGGGCGGCATTGAACTTGGTTGGCCAGGCATCGACGGAACCGACGCTTGCCAAGGC
                    ************************************************************

    Der f 22 cDNA   CACGGTCTTTCCTGTCCACTGACCAAAGGCCAGACCTACAATTACCACCTTGACTTTAAT
    Der f 22 gDNA   CACGGTCTTTCCTGTCCACTGACCAAAGGCCAGACCTACAATTACCACCTTGACTTTAAT
                    ************************************************************

    Der f 22 cDNA   CTCGGCGACGATGTACCAACA---------------------------------------
    Der f 22 gDNA   CTCGGCGACGATGTACCAACAGTGAGTTTATCCAGACAGTCAATTATGGCGATAAATAAA
                    *********************

    Der f 22 cDNA   ---------------------------------------GCTAACGTAACGGCCACAGTG
    Der f 22 gDNA   TGATTTATTCATTATTTTTTATTATTACACTTCACACAGGCTAACGTAACGGCCACAGTG
                                                           *********************

    Der f 22 cDNA   CGATTGGAAAACGGACATGGTGGCGACTTGCTTTGCGGCAGAATGCACATTAGTCTTCAA
    Der f 22 gDNA   CGATTGGAAAACGGACATGGTGGCGACTTGCTTTGCGGCAGAATGCACATTAGTCTTCAA
                    ************************************************************

    Der f 22 cDNA   AACTAA
    Der f 22 gDNA   AACTAA
                    ******
```

Supplementary Figure S1. Multiple alignments of cDNA and gDNA sequences of (A) Der f 2 and (B) Der f 22. Identical nucleotides at the same position are indicated by asterisks, and deletions of corresponding nucleotides are indicated by dashes. GT and AG boundaries of the intronic sequences are underlined.

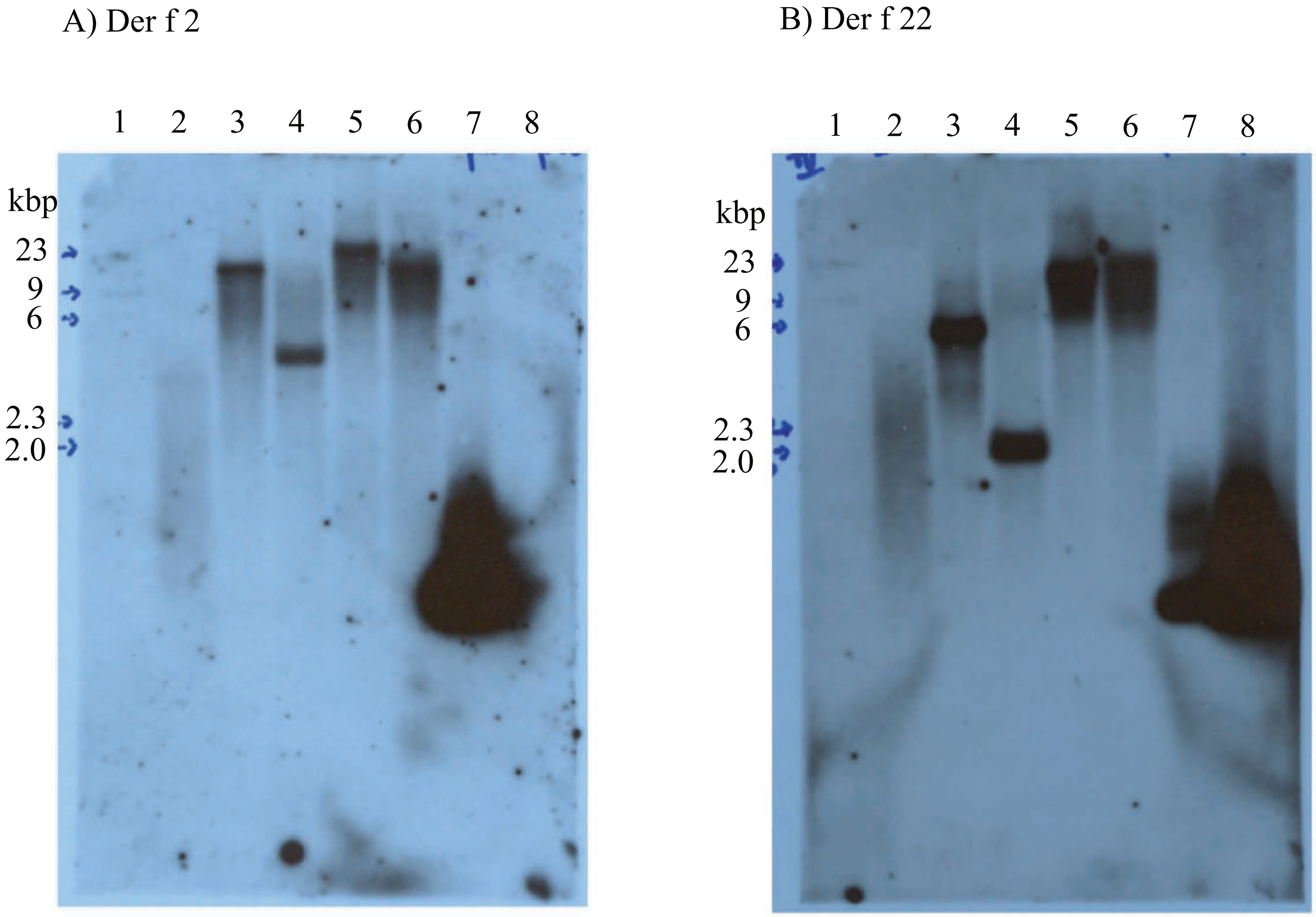

Key
1 - HindIV
2- Cla I
3- Eco RI
4- MscI
5- HindIII
6- BamHI
7- Der f 2 probe
8- Der f 22 probe

Supplementary Figure S2. Genomic Southern blot analysis of (A) Der f 2 and (B) Der f 22 using six restriction enzymes, HindIV (1), Cla I (2), EcoR I (3), Msc I (4), Hind III (5) and BamH I (6). After electrophoresis on a 1.0% agarose gel, the separated fragments were transferred to a Hybond-N membrane and hybridized with the DIG-labeled PCR amplified probes. Full length blots are presented.
